# Childhood tuberculosis and factors associated with mortality and loss to follow-up at a major paediatric treatment centre in Southern Ghana

**DOI:** 10.11604/pamj.2022.43.90.35440

**Published:** 2022-10-19

**Authors:** Adwoa Kumiwa Asare Afrane, Yakubu Alhassan, Vincent Ganu, Yaw Adusi-Poku, Bamenla Quarm Goka, Awewura Kwara

**Affiliations:** 1 Department of Child Health, Korle Bu Teaching Hospital, Accra, Ghana,; 2 Department of Child Health, University of Ghana Medical School, Accra, Ghana,; 3 Department of Biostatistics, School of Public Health, University of Ghana, Legon, Ghana,; 4 Infectious Disease Unit, Department of Medicine, Korle-Bu Teaching Hospital, Accra, Ghana,; 5 National Tuberculosis Control Programme, Ghana Health Service, Accra, Ghana,; 6 Department of Medicine, University of Florida College of Medicine, Gainesville, Florida, USA

**Keywords:** Childhood tuberculosis, treatment outcomes, pulmonary TB, children, Ghana

## Abstract

**Introduction:**

tuberculosis (TB) is a major cause of morbidity and mortality in children in low- and middle-income countries. This study described the clinical presentation and identified factors contributing to poor outcome of childhood TB at Korle Bu Teaching Hospital (KBTH), Accra, Ghana.

**Methods:**

this was a retrospective cohort study of children aged ≤ 14 years with TB registered for treatment at KBTH from 2015 to 2019. Treatment outcomes were recorded as treatment success and unsuccessful outcomes (died and loss to follow-up). Multivariable logistics regression was conducted to assess factors associated with an unsuccessful outcome.

**Results:**

of 407 children with TB registered during the period, 269 (66.1%) patients had pulmonary tuberculosis (PTB). Of the 138 patients with extra-pulmonary TB (EPTB), 68 (49.3%) had TB lymphadenitis. The TB/HIV coinfection rate was 42.8%. The overall treatment success rate was 68.3%, whilst 71(17.4%) died, and 58 (14.3%) were lost to follow-up. Factors associated with death were age below 1 year (AOR: 3.46, 95% CI: 1.48-8.10, p=0.004) and having HIV coinfection (AOR: 1.89, 95% CI: 1.04-3.43, p=0.037). Factors associated with loss to follow-up were age below 1 year (AOR: 2.91, 95% CI: 1.12-8.59, p=0.029) and having EPTB (AOR: 2.40, 95% CI: 1.24-4.65, p=0.009).

**Conclusion:**

childhood TB treatment success in our population was below the national target of 85%, with high mortality and loss to follow-up rates, especially in younger children and those with HIV coinfection or EPTB. Tailored treatment strategies may be needed for children at risk of unsuccessful treatment outcome, especially among infants.

## Introduction

Childhood tuberculosis (TB) remains a major public health concern as it contributes to significant child morbidity and mortality globally. According to the 2021 World Health Organization (WHO) global TB report, a total of 9.9 million people fell ill with TB in 2020 of which children younger than 15 years of age accounted for 11% [1]. In 2020, there were 1.3 million deaths from TB among HIV-negative people and 214,000 deaths among HIV -positive people. Of these deaths, 14% occurred among children under the age of 15. After decades of being in the shadow, the first Roadmap for Childhood TB was drawn by the WHO in 2013 with a goal of a world without TB deaths in children [2]. Subsequently, the WHO 2018 TB Roadmap has drawn Childhood TB further into the global spotlight, with a greater understanding of the challenges faced in addressing TB in children [1].

The actual magnitude of the childhood TB epidemic is difficult to assess, mainly due to diagnostic difficulties, poor access and delay in diagnosing and initiating appropriate TB treatment in children [3]. Another important challenge to global TB control is the human immunodeficiency virus (HIV) pandemic [4]. Children living with HIV face unique risks because of the higher rate of household exposure to TB, faster progression from infection to disease, and high incidence of other infections that may complicate diagnosis [4].

Enhanced analysis of Paediatric TB programme data is needed to maximize programme performance and improve treatment outcomes in various settings worldwide. Treatment outcome results serve as a proxy of the quality of treatment provided by a health care system [5]. The WHO sets targets for all National TB Control Programmes (NTPs) to successfully treat 85% of TB in order to interrupt the transmission, reduce mortality and prevent emergence of drug resistance [6]. Studies from sub-Saharan Africa have reported successful treatment outcomes for children ranging from 77.4% to 79.2% [7,8]. This suggests that at least 20% of children have suboptimal outcomes and there could be underlying clinical and demographic factors that may need to be considered in optimizing childhood TB treatment.

The NTP in Ghana leads the response to the fight against TB and continues to implement interventions according to the WHO Stop TB strategy [2]. High mortality rates prior to completion of anti-TB therapy have been observed in our clinic but no studies have been conducted to identify the contributing factors for the high death rates. The identification of the subgroup of patients at increased risk of death or default may be useful in designing additional measures to improve outcomes or areas for improvement in patient care. Thus, this study sought to assess the clinical presentation and treatment outcomes of childhood TB at the Korle Bu Teaching Hospital (KBTH) in Accra, Ghana. We also examined factors associated with death and loss of follow-up (LTFU) during anti-TB treatment in the study population with the aim of alerting the clinicians and NTP to the modifiable factors that may require additional or focused measures to improve overall TB treatment outcomes in children.

## Methods

**Study design and setting**: a retrospective cohort study of children aged ≥ 14 years with TB registered for treatment at the Department of Child Health in KBTH, Accra, Ghana from 1^st^ January 2015 to 31^st^ December, 2019 was conducted. Patients older than 15 years old treated at the centre were excluded from the study. KBTH is the biggest hospital in West Africa and the third largest on the African continent and has a bed capacity of about 2000 [9]. The Department of Child Health runs a 24-hour service for children aged 0-18 years and serves as a tertiary referral centre for other health facilities in the southern sector of Accra. At the Outpatient department (OPD) of the Child Health Department, patients diagnosed with TB are referred to the Public Health Unit (PHU) at OPD to be registered into the paediatric TB register. Paediatric patients diagnosed with TB in other departments of the KBTH hospital such as the Orthopaedic, and Paediatric Surgery are also referred to the Child Health Department to be registered for TB treatment. Tuberculosis diagnosis, treatment, and monitoring are done as per the NTP guidelines.

### Definition of terms

***Pulmonary Tuberculosis (PTB):*** patients with a documented diagnosis of pulmonary TB in the TB register for the period of assessment.

***Extrapulmonary TB (EPTB):*** patients with a documented diagnosis of extrapulmonary TB in the TB register for the period of assessment.

TB treatment outcomes are categorized according to National TB guidelines [10] in accordance with WHO guidelines as follows: ***cure:*** this is the proportion of patients among smear-positive patients that completed treatment and had at least two negative smears with an interval of at least 1 month, one of which should be obtained at the end of treatment; ***treatment completed:*** this is the proportion of patients that completed treatment, but sputum examination results are not available; ***died:*** the proportion of patients that died before the completion of treatment; ***lost to follow-up:*** this is the proportion of patients whose treatment was interrupted for two consecutive months or more; ***treatment failure:*** this is the proportion of patients who are still sputum smear positive at ≥5 months after the commencement of chemotherapy, or who interrupted treatment for ≥2 months after completing 1 month of chemotherapy, returned to treatment and are found to be smear positive; ***treatment success:*** defined as the sum of the cases that were cured and that completed treatment.

Clinical symptoms of pulmonary TB are a history of cough, fever, lethargy, poor weight gain and night sweats. The symptoms could also have been non-specific or persisted for more than 2 weeks following appropriate therapies for other conditions. Two samples are usually collected from children who can produce sputum for acid-fast bacilli (AFB) smear. If any of the sputum AFB tests are positive for TB, the patient is classified as sputum smear-positive pulmonary TB. Children whose smear results are negative are designated smear-negative TB. Other diagnostic tests like chest radiography and tuberculin tests are performed to aid the diagnosis of TB.

Presentation of EPTB depends on the sites of disease e.g. enlarged lymph nodes, spinal deformity (gibbus) or seizures. Extra-pulmonary TB is diagnosed after various relevant investigations have been conducted on samples obtained from children with suggestive clinical presentations. Examples of samples include cerebrospinal fluid for TB meningitis.

**Treatment of childhood TB and TB reporting System at Korle Bu Teaching Hospital:** the cost of treatment of TB in children in Ghana is free. Children diagnosed with TB have been treated with the standard 6 months´ regimen as per the NTP and WHO guidelines consisting of a 2-month intensive phase with 4 drugs (isoniazid, rifampicin, ethambutol and pyrazinamide) followed by 4 months continuation phase with isoniazid and rifampicin. WHO weight-band dosages for the antituberculosis drugs were used. For tuberculous meningitis and osteoarticular TB, the continuation phase is extended to a period of 10 months. At the end of the continuation phase, treatment outcomes are declared as successful or unsuccessful. Directly Observed Treatment Short Course Strategy (DOTS) is used in the management of TB. Each TB patient is routinely offered HIV testing by the healthcare workers at the DOTS facility. Patients diagnosed with TB/HIV co-infection are referred to the antiretroviral therapy (ART) clinic, where they are supposed to be offered ART within 2 weeks of the commencement of their anti-TB medications. The TB focal persons at the Department of Child Health are Public Health Nurses and they are responsible for registering and following up on TB patients. Details of patients treated for TB are entered into the TB paediatric register and updated regularly.

**Data collection and tool:** data were extracted from the Child Health TB register using a designed data extraction form. The register is a standardized document given by the NTP for use in hospitals in Ghana and is kept at the Public Health Unit at the Child Health Department. The data from the TB register is routine service delivery data for the purposes of programme monitoring and evaluation. Data extracted from the TB register included patients´ socio-demographic characteristics, type and category of TB, affected/ disease site, pre-treatment sputum smear examination results, HIV test results, and treatment outcome.

**Data handling and analysis:** data were abstracted from the TB register to a designed data extraction form and then entered into an Excel sheet. The data was edited, cleaned, and coded in Excel before being imported to Stata 16 IC (StataCorp, College Station, TX, USA) for analysis. Frequencies, proportions and summary statistics were used to describe the study population in relation to sociodemographic and clinical characteristics. The chi-square test and Fischer´s exact were used to assess the bivariate association between the socio-demographic and clinical characteristics and the various TB treatment outcomes observed in the study. Odds ratio and 95% CI were calculated using multivariate logistic regression analysis to identify independent associations for death and LTFU. All variables in the study were included in the multivariable logistic regression models. The type of patient (new or retreatment) was however excluded from the multivariable analysis because the smaller number of retreatment patients (n=4) all completed treatment which led to empty estimates from the logistic model. For all analyses, P values < 0.05 were considered significant.

**Ethical approval:** ethical approval was sought from the Korle Bu Scientific and Technical Committee (STC) as well as the Institutional Review Board (IRB). The approved study number is KBTH-STC 00015/2021. Permission was also sought from the Head of the Department of Paediatrics. The information from the study was used only for the purpose of the study and kept confidential. No personal identifiers were collected on the data extraction form.

## Results

### Demographic and clinical characteristics

During the five-year study period, the total number of cases per year was stable with a total of 407 children registering for TB treatment at the Child Health Department of KBTH. Their ages ranged from 2 months to 14 years with a mean (± standard deviation (SD)) age of 5.8 ± 4.2 years and 51.1% were males. Children, less than 5 years of age made up 42.8% of the patients. The prevalence or TB/HIV co-infection was 42.8% (Table 1). The majority of 403 (99.0%) of children diagnosed with TB were new cases with only 4 (1.0%) being retreatment cases. There was a total of 269 (66.1%) patients with pulmonary TB and 138 (33.9%) patients with EPTB. For those with pulmonary TB, the majority (93.3%, n=254/269) had clinically diagnosed pulmonary TB whilst 6.7% (n=18/269) had a sputum-positive result (Table 1).

**Table 1:** demographic and clinical characteristics and treatment outcomes of children with TB at the Korle-Bu Teaching Hospital, Accra from January 2015 to December 2019

		TB treatment outcomes			P- value
Characteristics	Total	Completed	Loss to follow-up	Died	
	n (%c)	n (%r)	n (%r)	n (%r)	
Total	407 (100.0)	278 (68.3)	58 (14.3)	71 (17.4)	
**Years**					<0.001
2015	90 (22.1)	67 (74.4)	5 (5.6)	18 (20.0)	
2016	81 (19.9)	56 (69.1)	7 (8.6)	18 (22.2)	
2017	79 (19.4)	47 (59.5)	14 (17.7)	18 (22.8)	
2018	71 (17.4)	55 (77.5)	6 (8.5)	10 (14.1)	
2019	86 (21.1)	53 (61.6)	26 (30.2)	7 (8.1)	
**Age group**					0.010
<1 year	59 (14.5)	30 (50.8)	11 (18.6)	18 (30.5)	
1-4 years	115 (28.3)	75 (65.2)	22 (19.1)	18 (15.7)	
5-9 years	129 (31.7)	97 (75.2)	11 (8.5)	21 (16.3)	
10-14 years	104 (25.6)	76 (73.1)	14 (13.5)	14 (13.5)	
**Sex**					0.97
Female	199 (48.9)	136 (68.3)	29 (14.6)	34 (17.1)	
Male	208 (51.1)	142 (68.3)	29 (13.9)	37 (17.8)	
**Type of TB**					0.082
Extra pulmonary	138 (33.9)	89 (64.5)	29 (21.0)	20 (14.5)	
Clinically diagnosed	251 (61.7)	176 (70.1)	27 (10.8)	48 (19.1)	
Sputum positive	18 (4.4)	13 (72.2)	2 (11.1)	3 (16.7)	
**Type of extra pulmonary TB (N=138)**					<0.001
Abdomen	11 (8.0)	6 (54.6)	4 (36.4)	1 (9.1)	
Bone	2 (1.4)	1 (50.0)	1 (50.0)	0 (0.0)	
Disseminated	1 (0.7)	0 (0.0)	0 (0.0)	1 (100.0)	
Lymphadenitis	68 (49.3)	51 (75.0)	11 (16.2)	6 (8.8)	
Meningitis	18 (13.0)	3 (16.7)	7 (38.9)	8 (44.4)	
Pleural effusion	7 (5.1)	4 (57.1)	1 (14.3)	2 (28.6)	
Spine	31 (22.5)	24 (77.4)	5 (16.1)	2 (6.5)	
**HIV status**					0.004
HIV negative	233 (57.2)	160 (68.7)	42 (18.0)	31 (13.3)	
HIV positive	174 (42.8)	118 (67.8)	16 (9.2)	40 (23.0)	
Type of patient					0.39
New	403 (99.0)	274 (68.0)	58 (14.4)	71 (17.6)	
Retreatment	4 (1.0)	4 (100.0)	0 (0.0)	0 (0.0)	

%c: column percentage. %r: row percentage

### Treatment outcome of the study participants

The treatment outcome of the 407 study patients assessed showed that overall, 278 (68.3%) of them completed treatment, 58 (14.3%) were lost to follow-up and 71 (17.4%) died (Table 1). The treatment success rate fluctuated during the study period. The trend of unsuccessful outcome (death, and loss to follow-up) consistently increased progressively over the years from 25.6% in 2015 to 40.5% in 2017. However, the rate decreased in 2018 to 22.5% and then increased to 38.3% in 2019.

### Factors associated with treatment outcome

There was a significant association between the age group and TB treatment outcome (p=0.008). The percentage of completed treatment was highest among older children (10-15 years) with 73.1% completed treatment and lowest (50.8%) among less than 1-year-old children. The percentage of death was higher among children less than (30.5%) compared to the other age groups (Table 1). The site of EPTB was found to be significantly associated with TB treatment outcome with a high completed treatment rate in children with lymphadenitis (75.0%) and spine (77.4%) (p<0.001). Although, the completed treatment rate was similar between HIV-negative (68.7%) and HIV-positive (67.8%) TB patients, the mortality among the children with HIV (23.0%) was almost twice that among those without HIV infection (13.3%) (p=0.004) (Table 2). Overall, 40 (56.3%) of the 71 participants who died had HIV coinfection (Table 1).

**Table 2:** univariable and multivariable logistic regression analyses showing the factors associated with mortality among children with TB at KBTH from 2015 to 2019

		TB treatment outcomes		Binary logistic regression models of factors associated with mortality among children on TB treatment			
	Total	Completed treatment	Death	Univariable analysis		Multivariable analyses	
Variables & categories	N	n (%)	n (%)	Unadjusted Odds Ratio [95% CI]	P-value	Adjusted Odds Ratio [95% CI)	P-value
**Total**	349	278 (79.7)	71 (20.3)				
**Year**							
2015	85	67 (78.8)	18 (21.2)	1.00 [reference]		1.00 [reference]	
2016	74	56 (75.7)	18 (24.3)	1.20 [0.57, 2.52]	0.636	1.09 [0.50, 2.36]	0.830
2017	65	47 (72.3)	18 (27.7)	1.43 [0.67, 3.02]	0.356	1.55 [0.71, 3.37]	0.267
2018	65	55 (84.6)	10 (15.4)	0.68 [0.29, 1.59]	0.369	0.65 [0.27, 1.56]	0.335
2019	60	53 (88.3)	7 (11.7)	0.49 [0.19, 1.26]	0.141	0.53 [0.20, 1.39]	0.195
**Age group**							
<1 year	48	30 (62.5)	18 (37.5)	3.26 [1.44, 7.37]	0.005	3.39 [1.42, 8.09]	0.006
1-4 years	93	75 (80.6)	18 (19.4)	1.30 [0.60, 2.81]	0.499	1.46 [0.65, 3.28]	0.359
5-9 years	118	97 (82.2)	21 (17.8)	1.18 [0.56, 2.46]	0.669	1.23 [0.58, 2.63]	0.591
10-15 years	90	76 (84.4)	14 (15.6)	1.00 [reference]		1.00 [reference]	
**Sex**							
Female	170	136 (80.0)	34 (20.0)	1.00 [reference]		1.00 [reference]	
Males	179	142 (79.3)	37 (20.7)	1.04 [0.62, 1.76]	0.876	0.90 [0.52, 1.57]	0.710
**Type of TB**							
Extrapulmonary TB	109	89 (81.7)	20 (18.3)	0.83 [0.47, 1.48]	0.533	1.16 [0.58, 2.31]	0.667
Pulmonary TB	240	189 (78.8)	51 (21.2)	1.00 [reference]		1.00 [reference]	
**HIV status**							
HIV negative	191	160 (83.8)	31 (16.2)	1.00 [reference]		1.00 [reference]	
HIV positive	158	118 (74.7)	40 (25.3)	1.75 [1.03, 2.96]	0.037	1.89 [1.02, 3.49]	0.042

COR: crude odds ratio. AOR: adjusted odds ratio. CI: Confidence interval. P-value notation: *: p<0.05. **: p<0.01. ***: p<0.001.

### Factors associated with death and loss to follow-up in multivariate analysis

In the multivariable analysis, children below of age were over three times more likely to die as compared to children aged 10 -15 years (AOR: 3.39, 95% CI: 1.42-8.09, p=0.006). Also, children who are positive for HIV were almost two times more likely to die as compared to HIV-negative children (AOR: 1.89, 95% CI: 1.02-3.49, p=0.042) (Table 2). Compared to children diagnosed in 2015, loss to follow-up was about 4 times higher for children diagnosed in 2017 (AOR: 3.93, 95% CI: 1.26-12.21, p=0.018) and over 8 times higher in 2019 (AOR: 8.34, 95% CI: 2.84-24.43, p<0.001). Children below of age were four times more likely to be lost to follow-up as compared to children aged 10-15 years. (AOR: 4.51, 95% CI: 1.63-12.50, p=0.004). Children with EPTB were over two times more likely to be lost to follow-up as compared to pulmonary TB patients (AOR: 2.55, 95% CI: 1.25-5.21, p=0.010) (Table 3).

**Table 3:** univariable and multivariable logistic regression analyses showing the factors associated with loss to follow-up among children with TB at KBTH from 2015 to 2019

		TB treatment outcomes		Binary logistic regression models of factors associated with LTFU among children on TB treatment			
	Total	Completed treatment	LTFU	Univariable analysis		Multivariable analyses	
Variables & categories	N	n (%)	n (%)	Unadjusted Odds Ratio [95% CI]	P-value	Adjusted Odds Ratio [95% CI)	P-value
**Total**	336	278 (82.7)	58 (17.3)				
**Year**							
2015	72	67 (93.1)	5 (6.9)	1.00 [reference]		1.00 [reference]	
2016	63	56 (88.9)	7 (11.1)	1.68 [0.50, 5.57]	0.400	1.92 [0.55, 6.65]	0.304
2017	61	47 (77.0)	14 (23.0)	3.99 [1.35, 11.84]	0.013	3.93 [1.26, 12.21]	0.018
2018	61	55 (90.2)	6 (9.8)	1.46 [0.42, 5.05]	0.548	1.55 [0.43, 5.56]	0.505
2019	79	53 (67.1)	26 (32.9)	6.57 [2.36, 18.28]	<0.001	8.34 [2.84, 24.43]	<0.001
**Age group**							
<1 year	41	30 (73.2)	11 (26.8)	1.99 [0.81, 4.87]	0.132	4.51 [1.63, 12.50]	0.004
1-4 years	97	75 (77.3)	22 (22.7)	1.59 [0.76, 3.34]	0.219	2.09 [0.93, 4.73]	0.075
5-9 years	108	97 (89.8)	11 (10.2)	0.62 [0.26, 1.43]	0.260	0.78 [0.32, 1.93]	0.589
10-15 years	90	76 (84.4)	14 (15.6)	1.00 [reference]		1.00 [reference]	
**Sex**							
Female	165	136 (82.4)	29 (17.6)	1.00 [reference]		1.00 [reference]	
Males	171	142 (83.0)	29 (17.0)	0.96 [0.54, 1.69]	0.881	0.96 [0.52, 1.78]	0.898
**Type of TB**							
Extrapulmonary TB	118	89 (75.4)	29 (24.6)	2.12 [1.20, 3.77]	0.010	2.55 [1.25, 5.21]	0.010
Pulmonary TB	218	189 (86.7)	29 (13.3)	1.00 [reference]		1.00 [reference]	
**HIV status**							
HIV negative	202	160 (79.2)	42 (20.8)	1.00 [reference]		1.00 [reference]	
HIV positive	134	118 (88.1)	16 (11.9)	0.52 [0.28, 0.96]	0.038	0.60 [0.28, 1.25]	0.173

**COR:** crude odds ratio. **AOR:** adjusted odds ratio. **CI:** Confidence interval. **NOTE:** The transferred case was excluded from this analysis.

### Antiretroviral therapy initiation and TB treatment outcome

Amongst the 174 HIV-positive patients, 100 of them had records of when they started ART. Among these 100 patients, 66% (66/100) of them started ART after TB treatment. The median days to start TB treatment after initiation of ART was 415 days (IQR: 93 to 1246 days) whilst the median days to initiate ART after the start of TB treatment was 104 (IQR: 47 to 191 days). Mortality was higher among HIV-positive patients that started ART before TB treatment (20.3%) compared to the HIV-positive patients that started ART treatment after TB treatment (3.0%) (p=0.005).

## Discussion

In this study, we described the characteristics of childhood TB and identified factors associated with death and loss to follow-up over a 5-year period. Understanding the local burden and its contribution to overall disease is an essential tool for achieving global reduction of TB [11]. Overall the successful treatment outcome was below the global target of 85%. Treatment outcome at the end of therapy is an important marker of the success of TB control programmes [12]. While the overall proportion of children with successful outcome was below the global target of 85%, it corresponds with childhood TB outcomes in similar settings [1]. The successful treatment outcome of 68.3% from our study is lower than reports from countries like Ethiopia (85%) [13], Nigeria (77%) [14] and Malawi (77%) [15] in the African Region. Factors associated with death were age below and having HIV coinfection. Factors associated with loss to follow-up were age below and having EPTB.

Pulmonary TB was the most common form of TB with a low proportion of smear-positive TB cases. The finding of the low proportion of patients with sputum-positive PTB is in line with other studies, [14-16] and highlights the challenges of specimen collection and microbiological confirmation in childhood TB. Although bacterial confirmation remains the cornerstone of TB diagnosis, it is difficult to confirm TB in children because children are mainly paucibacillary in nature and cannot expectorate. Diagnosis in children as in our study is mostly based on history and clinical presentation [17]. The observed proportion of EPTB in the study (33.9%) was higher than that obtained in a previous study by Ohene *et al*. (17.7%) also done in Ghana [18]. Studies done in Benin [19], Turkey [20] and Cote D´Ivoire [14] also obtained rates lower than that in our study (9-23%). The proportion of EPTB in our study could be due to the higher number of older children in our study in whom EPTB is more common [3]. The finding of lymph node disease being the most commonly affected extrapulmonary site was also consistent with other studies [13,21-23].

Death and loss to follow-up occurred in 17.4% and 14.3% of our study population, respectively. The mortality rate in our study is higher than other studies from other African countries such as Ethiopia (3.3%) [24], Botswana (10.5%) [25] and Tanzania (10.9%) [26] but similar to that in Malawi (17%) [24]. In our study, there was higher mortality in children younger than 5 years. This is in agreement with a study in Malawi which reported a decline in the death rate with increasing age [24]. Our multivariate analysis identified age < 1-year-old to be associated with mortality. These children died within a month of starting treatment. It is more challenging to diagnose TB in the younger age group and diagnosis is usually clinical thus delay in starting treatment is common.

We found a high prevalence of HIV infection among TB patients (42.8 %). This rate is higher than the current global estimate of 15%, as well as estimates from the African Region (32-36%) and the estimated national average of 24% [1]. The rate obtained in our study is also higher than a study by Adejumo *et al*. in Togo (29%) which used programmatic data and Segbedji in Nigeria (14.9%) which used multicenter data [15,27]. The observed variation might be due to an improvement in the quality of services in the TB/HIV clinics. In our setting, every TB patient was tested for HIV in accordance with WHO guidelines.

The LTFU rate in this study showed an increasing trend over the 5-year review and was relatively high (17.3%) as compared to that of the national average in Ghana of 4.1% and the global estimate of 6% [1]. Independent factors associated with loss to follow-up were an age of less than one year of age and patients with EPTB and this would have to be examined further. It is possible that patients with EPTB were more likely to have been referred from other facilities and hence returned back to their facility for treatment and this may have been captured as a loss to follow-up. Considering mortality was high amongst infants, it is possible that some of the LTFU died before their follow-up date. More efforts must be made by public health nurses in charge of following up with patients to ensure that patients are routinely called to ensure that they adhere to their visits and to verify reasons for the LTFU. In our study, the absence of data on microbiologic confirmation in most diagnosed patients is a limitation and as such further emphasizes the need for the development of new diagnostics for childhood TB. Mortality from alternative diagnoses as possible, and the exact causes of death were unknown. Strengthening national surveillance to capture all diagnosed cases of TB in children, and providing training or guidance to healthcare providers to record more detailed data about childhood TB cases will help provide more accurate estimates of the national disease burden and potentially increase political commitment to address childhood TB.

## Conclusion

The successful treatment outcome was below the NTP target of 85% because of high mortality and LTFU rates. Our findings suggest that HIV coinfection and children less than one year were at higher risk of death and LTFU. There is a need for a renewed focus on improving TB outcomes in younger children, those with HIV coinfection and those with EPTB. Strategies to improve TB treatment outcomes include synergizing services for TB and HIV, the development of more reliable tests for diagnosis in children and enhanced follow-up. Further studies should be conducted to identify the actual cause of death of TB patients.

### What is known about this topic

- Tuberculosis (TB) is a major cause of morbidity and mortality in children in low- and middle-income countries;
- The WHO sets targets for all National TB Control Programmes (NTPs) to successfully treat 85% of TB in order to interrupt the transmission, reduce mortality and prevent the emergence of drug resistance.

### What this study adds

- Factors associated with death were age below and having HIV coinfection;
- In the children with TB/HIV coinfection, there was a delay in starting ART after starting TB treatment;
- Factors associated with loss to follow-up were age below and having EPTB.
